# Henry Tye: the future of theoretical physics and advice to young researchers

**DOI:** 10.1093/nsr/nwab074

**Published:** 2021-04-28

**Authors:** Yi-Fu Cai, Sheng-Feng Yan

**Affiliations:** Department of Astronomy, School of Physical Sciences, USTC; post-doc fellow, National Institute for Nuclear Physics in Milan

## Abstract

Professor Henry Tye (戴自海) is a world-renowned expert in theoretical particle physics, string theory and cosmology. He was recently the IAS Professor at the Jockey Club Institute for Advanced Study (IAS) and the Hong Kong University of Science and Technology (HKUST), and is the Horace White Professor of Physics (Emeritus) at Cornell University. He has a lot of experience in research status in both China and the United States. Recently, *NSR* invited Professor Yi-Fu Cai (蔡一夫) from the University of Science and Technology of China (USTC) to interview Prof. Tye on his personal views on the future of theoretical physics, his own experience, and his advice to young researchers.

## String theory and the beginning of our universe

***Cai:*** It is well known that you are an expert in the field of string theory, string phenomenology and string cosmology. How does string theory describe the very beginning of our universe?

***Tye:*** We started from nothing, not even space. If there's no space, the definition of time is also a bit uncertain. Out of nothing, a quantum fluctuation popped out, then the universe inflated. It gives us the inflationary universe and the rest of the universe follows.

About the details of this, many people have different opinions. The question is that, if it's fluctuation from nothing, what fluctuation has a reasonable probability? We tried to do some work on this, but are still not clear because more details are necessary. Our idea is that it fluctuates to a brane world scenario, which means the fluctuation prefers some dimensions compactified and some other dimensions forming the observed world, and then there's new phenomenon associated with that. That's how the universe began. But that part is very speculative.

After the very beginning, string theory naturally suggests brane inflation, which sometimes was specifically the brane-antibrane inflation. It's naturally suggested by most scenarios of string theory. From that, our universe follows.

Once we take that picture, it automatically means that today our universe is really described by a brane world picture, meaning that we live in a three-dimensional brane, and outside the brane is the so-called bulk, which are the six extra dimensions. That's the picture that follows brane inflation and tells us what our universe looks like today.

The early universe and the later universe combined together—it seems to be roughly the picture that quite a number of people in the community are happy to accept. Of course, many details of the calculation should be worked out before we know whether this is really a good picture or not.

**Figure 1:**
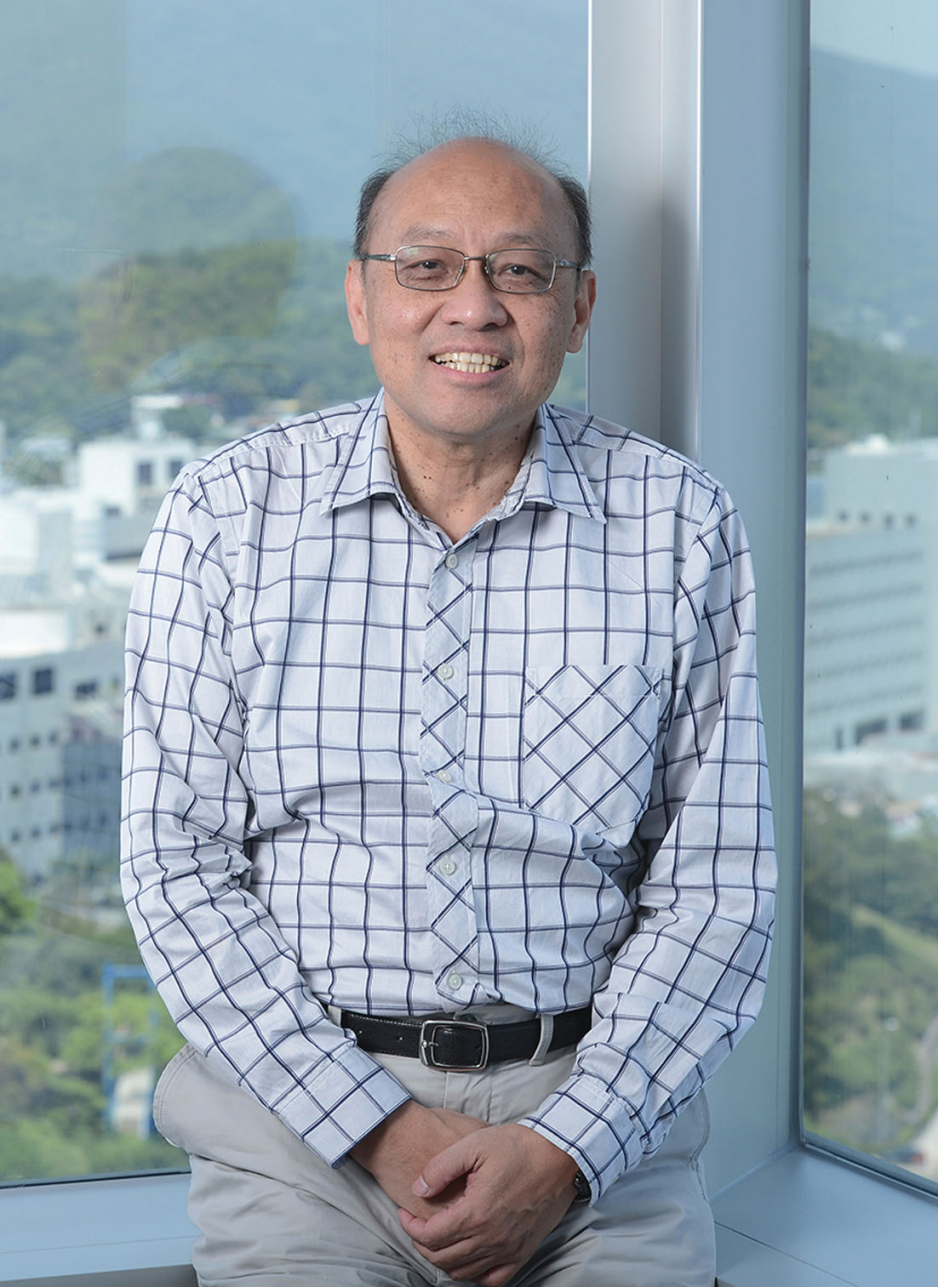
Professor Henry Tye is a world-renowned expert in theoretical particle physics, string theory and cosmology *(Courtesy of Prof. Tye)*.

***Cai:*** Do you think observation could be the bottleneck for string theory?

***Tye:*** I don’t think so. Some of the questions can be answered by observation. The simplest one is cosmic strings. People are searching for that, either from gravitational waves or otherwise. If they discover cosmic strings and study them, it will be the smoking gun for string theory. We know all particles, such as electrons or photons, are tiny little bits of string. But some strings, originated from the early universe, are big. There would

[The brane world picture means] that we live in a three-dimensional brane, and outside the brane is the so-called bulk, which are the six extra dimensions.

—Henry Tye

be some strings stretching across the sky, as big as the universe. They are the cosmic strings.

There's a chance that scientists can find them, maybe in the next five to ten years. There have been people saying that they have seen something like cosmic strings. But the evidence is not good enough. This is one detection that can put string theory on a firm ground.

***Cai:*** Do scientists in string theory do some observations themselves, or collaborate with experimentalists, or just wait for the possible results from the experimentalists?

***Tye:*** It is easy to do observations of cosmic strings. You just need to focus on current astrophysical effects, so astrophysicists who do observations are able to look for them. They don’t need to know string theory, they just need string theory to tell them where to look and what to look for. But other experiments in string theory are not so easy to carry out. So, I think a little bit more interaction between theorists and experimentalists is important.

## String theory and cosmology

***Cai:*** I’m always positive about string theory. If, in five or ten years, we have some amazing things happening and string theory verified—I don’t know if it's possible—how would the verified string theory impact physical science?

***Tye:*** I’ve been working on one problem that I want to understand: why the cosmological constant or the dark energy is so small. Another parameter which looks very small is the Higgs boson mass—why its mass is not dictated by the gravitational (i.e. Planck) scale. Sometimes it's called the mass hierarchy problem. To understand it, people tried many scenarios. They found that the most convincing scenario is supersymmetry—if you take a tree-level Higgs mass to be of the order of 100 GeV, if the supersymmetry breaking scale is of the order of 100 GeV or a few hundred GeV, the quantum corrections to the Higgs mass will be a few hundred GeV. From that point of view, the Higgs mass is technically natural. That means that it's stable under quantum corrections. From that it requires supersymmetry. And from supersymmetry, it requires super-particles. One of the super-particles is dark matter, and people are searching for dark matter. This has been the mainstream of particle physics in the last 40 years. But it does not answer why the tree-level mass is not much bigger. So, the Higgs boson mass is not yet natural, only technically natural.

Now, I come to the other problem, which is the dark energy and the cosmological constant. This problem is much more serious than the Higgs boson mass. Higgs boson mass requires maybe 60 orders of magnitude fine-tuning. But the cosmological constant requires 120 orders of magnitude fine-tuning. Why don’t people study that? It's very clear that you can calculate it in Einstein's theory. But Einstein's theory allows you to introduce the cosmological constant by hand. You can always fine-tune it to agree with today's nature, but it lacks explanation because of the 120 orders of magnitude of fine-tuning.

So now, if you want to understand the cosmological constant problem, you have to go to string theory. Because in quantum field theory in the framework of Einstein, it's a parameter in the model. But in string theory, you can calculate that, and look for regions where that value is naturally small. I think the future goal is to understand why string theory can yield a naturally small cosmological constant.

***Cai:*** Related to this vacuum energy, which is an extremely small number, there is a cosmological conception that our universe has experienced inflation, and in inflation the vacuum energy is pretty high. So, is it possible that these two are actually the same thing?

***Tye:*** That's a very good question. In fact, I think this is one of the key hints as to what's going on. In the early days, before people knew enough, they said, maybe the vacuum energy is zero, due to some unknown symmetry. Hawking and Coleman proposed that the smallness was because there is a probability distribution that perfects zero.

Once you have inflation, those ideas don’t hold anymore. Because if you create a universe that started with a very small cosmological constant, then you have no inflation. The existence of inflation tells you that it should be naturally small dynamically, not by some other way. Inflation actually tells you that it has to do with dynamics, not symmetry. So, whatever you want to do to solve the problem, that solution has to allow the early universe to have a large vacuum energy that drives inflation. It's no longer the case that you can fine-tune it at the beginning of the universe.

***Cai:*** You described the theoretical interpretation of the natural smallness of the cosmological constant. And also, there are hints from the inflationary cosmology that it might be dynamical. I believe this definitely is your current research topic. Are there any other recent research topics that you want to share with us?

***Tye:*** I, with my collaborators, are trying to finish a piece of work that is applying the idea of a naturally small constant to cosmology. I’m really a bit nervous because we have big claims. We use the string theory motivated model to solve some of the major cosmological problems in nature, which are the Hubble tension, the lithium problem during nucleosynthesis, the weak lensing issue with regard to the clustering, and the mass hierarchy.

## Fundamental science calls for international collaboration

***Cai:*** You have been spending most of your time in the US. Now you’re putting huge effort into developing fundamental science research in HKUST. Can you make some comments about the impact of such an international experience on you? And today there is some competition between China and other countries. How do you effectively promote international cooperation when facing the increasingly fierce international competition of science and technology?

***Tye:*** I was born in Shanghai. I grew up in Hong Kong since I was a baby and went to school there. Then I went to the United States. When I finished my education, there was nobody working in my profession in Hong Kong and no job on the Chinese mainland either. Thus, I stayed on in the United States. After a few decades, my mother and my wife's mother were getting old, so we decided to come back to Hong Kong to spend some time with them.

At this time, China was really coming up very strong and very fast, and I really hoped that Hong Kong could play a role as a bridge between East and West, not only for fundamental physics, which nobody in Hong Kong was working on and which I wanted to build up, but also for science and technology as a whole. The first few years looked pretty good and promising, however, it's been disappointing in the most recent couple of years.

For academic communications, I feel that fundamental physics really has no border. Physicists with very different backgrounds are welcome to talk and discuss science, and to collaborate. We don’t care about their religion, the country in which they were raised, or their gender. Science communication is really one thing that can spread worldwide and connect everybody.

Now actually, Asia is coming up. Not only China, but also Japan, South Korea and maybe India all want to spend more effort in basic science. So, I do think that Asia has a bright future. For basic science, not only physics, but also life sciences and other areas, I hope that academic communications, interactions and exchanges will not suffer after the pandemic.

***Cai:*** Personally, I always believe that communication, cooperation and joint effort in fundamental science can always be a good window for people to know each other, and to try to solve some problems.

***Tye:*** I totally agree with you. By having more collaboration and interaction across countries, races and genders, we will know each other better, understand each other better, and that would certainly support the peace of the world. We have to work together to change all these things. I think country-to-country competition is good, but country-to-country fighting is not good. I think we are living in one world, we have to work together.

## Advice to young researchers

***Cai:*** Would you give some suggestions to the younger generation in the field of fundamental science, including string theory, cosmology and any other field within general fundamental science?

***Tye:*** My personal feeling is that for many people who are doing solid, good science, they stay in one area for their whole life. I think it's important to continue to learn and improve yourself.

At Cornell, we lived under the umbrella of some prominent people (especially Hans Bethe) who really knew how to appreciate young people.

—Henry Tye

If you stop learning when you are 25 years old, and continue working on the area of your thesis, even if you’re very lucky and your area is good, your development will slow down. If you learn physics for 10 years, and you continue to be curious and learn at my age—in your 70s—that will really benefit you over your lifetime. That will give you more ideas for research, give you a better perspective, and also, you will enjoy your work more, through interaction with more people.

Young people should be curiosity-driven, and should not worry too much about publication. I know that's important, but a lot of time people (understandably) pay too much attention to publication.

***Cai:*** They feel a lot of stress.

***Tye:*** Yes, I understand they have a lot of pressure. Actually, whatever kind of ideas you have, you can write it into an article. But if you have to publish a lot, you really have no time to sit back and think. I think the pressure to publish is not only in China, but also in the US and everywhere. So maybe society doesn’t need to emphasize publication as much.

***Cai:*** If that becomes true, it will relieve a lot of pressure on young researchers.

***Tye:*** Yes. But if you’re thinking about deep problems and have no publication yet, it's easy for the outside people to say you’re lazy. But some problems do take years to move over, to nurture, and to slowly get to the point.

I’m lucky that I came from Cornell, spent many years there. Before my time, Feynman was there. Feynman came from Los Alamos, and for a year or so had no result. Then he told the director of the lab, ‘You hire me, pay me well, however, I have nothing.’ And the director told him, ‘Don’t worry about publication. Just think what's interesting to you, and think about the problems that you are interested in.’

Later, in 1963, Ken Wilson came to Cornell. He had almost no publications by the time his tenure came up. He had only two papers of which he was not the senior author. But in the faculty meeting in 1969, they decided to promote him to full professor with hardly any publications. He tried to solve a deep problem, tried to understand what quantum field theory was.

Cornell really encouraged the young people to handle the important problems. At Cornell, we lived under the umbrella of some prominent people (especially Hans Bethe) who really knew how to appreciate young people. So senior people really have an impact on the young people. I think other places should do the same thing. But now, unfortunately, society, including US society, does not accept that you are working very hard if you have no publications for a few years.

***Cai:*** Nowadays, I have such a strong feeling that all of the young scientists are working very hard. They work hard to publish not only for their own future, but also for their students, who need publications to get their PhD degree. We want to make a better future for the even younger generation. I can’t say that the current environment does not allow us to think of the deep questions, but obviously it's not enough. We are really working hard and trying to build a beautiful land for the future.

***Tye:*** The system keeps changing. I remember that the first time I went to the Chinese mainland, the number of publications was important. People who have one piece of work would split it into multiple papers. Then, the quality of the publication became important, especially in chemistry or life sciences. They put three projects into one paper so that the paper was of a high quality. I think it's a big step forward. But I hope that the system will further mature to a stage where publication is unimportant compared to the really important projects.

I know it's very hard. But I think for China this change could be easier than for the US, because in the United States the salary of scientists depends on their grant, and their grant depends on their publication. But in China, salary does not depend on grants. So, in some sense, I think Chinese researchers may have less pressure.

But of course, some pressure may be unavoidable, just to prevent people from relaxing too much. There should be a good balance, and how to find the balance depends on the individual institution and research area.

